# Effect of Different Frequencies of Dental Visits on Dental Caries and Periodontal Disease: A Scoping Review

**DOI:** 10.3390/ijerph20196858

**Published:** 2023-09-28

**Authors:** Najith Amarasena, Liana Luzzi, David Brennan

**Affiliations:** Australian Research Centre for Population Oral Health, Adelaide Dental School, Faculty of Health and Medical Sciences, The University of Adelaide, Adelaide, SA 5005, Australia; liana.luzzi@adelaide.edu.au (L.L.); david.brennan@adelaide.edu.au (D.B.)

**Keywords:** frequency of dental visits, dental caries, periodontal disease, recall intervals, scoping review

## Abstract

Recommending dental visits every six months is commonplace among dental practitioners worldwide. A scoping review was conducted by electronically searching PubMed, Scopus and Embase to identify and map the nature of evidence for the effect of different frequencies of dental visits on dental caries and periodontal disease. Studies were written in English on the frequency of dental visits and published between January 2008 and April 2023. Three systematic reviews that evaluated the risk of bias, strength of studies and certainty of evidence were included from the 4537 articles yielded through the search strategy. The available evidence was weak and of low quality for the currently recommended frequencies of dental visits, whether these are fixed or universal. For adults, there was little to no effect of making biannual, biennial or risk-based dental visits on dental caries and periodontal disease, which was supported by moderate- to high-certainty evidence. Accordingly, it is suggested that dental professionals and dental insurance providers make individually tailored, customised and risk-based recommendations for dental visits, rather than encouraging fixed or universal frequencies of dental visits. For children and adolescents, further research on this issue warrants well-designed randomised controlled trials (RCTs) and cohort studies of sufficient duration with an adequate number of participants.

## 1. Introduction

Oral diseases, including dental caries, periodontal disease and oral cancer, are the most widespread, non-communicable diseases among humans, afflicting nearly 3.5 billion people globally [1,2]. The overwhelming case number of oral diseases, which exceeds that of all five main non-communicable diseases by approximately one billion, further reflects this [2]. Dental caries and periodontal disease are the most common oral diseases, with untreated dental decay being the most prevalent entity, affecting nearly 2.8 billion people globally [1], whereas 743 million were afflicted with severe periodontal disease, which was ranked as the sixth-most prevalent disease in the world [1,3]. According to the latest reports [2], the global prevalence of untreated dental caries in the primary dentition and permanent dentition across the WHO regions was 42.7% (range: 38.6–46.2%) and 28.7% (range: 25.4–33.6%), respectively, while the proportion of people with severe periodontal disease was 18.8% (range: 16.3–22.8%) in 2019 (Table 1).

Routine dental visits are important for the prevention and control of oral diseases, particularly the ubiquitous dental caries and periodontal diseases. This is to maintain good oral health given that such visits provide dental practitioners with an opportunity for the early detection and management of oral diseases in people of across all life stages [4,5,6,7,8]. It has been shown that people who make routine dental visits (routine attenders) have a better self-reported oral health with lower levels of dental caries and fewer teeth missing due to caries than problem-oriented visitors who attend sporadically for dental problems only [8,9,10]. Likewise, the non-regular dental visiting trajectories of dental patients aged 15–32 years has been associated with a lower self-rated oral health [10], while long-term routine dental attendance has been associated with lower levels of tooth loss and improved oral-health-related quality of life in older adults [11]. It is also noteworthy that making routine dental visits can be hampered by financial and non-financial barriers to obtain dental care [12,13,14]. Financial barriers, including avoiding or delaying dental visits as well as not obtaining recommended dental treatment due to cost and difficulty paying a dental bill, can be regarded as the main obstacles for seeking dental care, which in turn reflect the affordability and hardship in purchasing dental care [12,13]. Additionally, non-financial barriers, such as accessibility to and availability of dental services, oral health literacy and dental anxiety, can hinder one’s ability to make routine dental visits [14]. Encouraging patients to attend an oral health check-up every six months has become common practice among dental practitioners worldwide, while frequencies such as once in 3, 12 or 24 months are less common. However, deliberation among oral health researchers on the evidence-based status of six-monthly dental visits has been continuing since 1970s [15]. Moreover, there is a paucity of evidence for or against recommending any of these frequencies [4,5,6].

The National Oral Health Plan 2004–2013 in Australia documented the importance of promoting oral health and adhering to consistent as well as evidence-based oral health messages [16]. With the aim of reaching a national consensus on oral health messages for the public, a workshop was held in 2009 [17]. The main professional organisations and researchers involved in the field of oral health reviewed and discussed the literature to formulate a set of 11 oral health messages, which was coherent with general health messages. One of these messages was on the frequency of oral health visits and, based on the available evidence, it was recommended that people should consult their oral health professionals about their individual risk level and how frequently they should ideally visit for an oral health check.

It has been a little over a decade since the development of these oral health messages, despite Australia’s National Oral Health Plan 2015–2024 emphasising that these messages should be reviewed regularly [18]. In the meantime, several studies have been conducted to discern whether any particular frequency of visiting for an oral health check is more effective than another. Against this background, conducting a scoping review was deemed appropriate and timely. Accordingly, the objective of this scoping review was to identify and map the nature of the evidence for the effect of different frequencies of dental visits on dental caries and periodontal diseases, as reported in the literature.

## 2. Materials and Methods

This scoping review was based on scoping review methodological frameworks published elsewhere [19,20,21]. Compared with systematic reviews, scoping reviews have more inclusive and exploratory study designs [19,20,21]. While encompassing numerous studies to map and/or identify the gaps in the current evidence on a given topic, scoping reviews concentrate on breadth instead of detail. Scoping reviews employ narrative analytical techniques and are typically not designed to assess the quality of individual studies as opposed to the synthesis and/or aggregation of the quantitative data methods used in systematic reviews [19,20,21].

### 2.1. Scoping Review Question

Our scoping review, including the search strategy, was based on the following broad question:

What evidence is currently available for the effect of different frequencies of dental visits on dental caries and periodontal disease?

### 2.2. Dental Caries

Dental caries is a chronic disease with a localised destruction of tooth enamel and dentine caused by acids that are produced as a result of cariogenic biofilm acting on fermentable carbohydrates [22]. A dynamic process of demineralisation and remineralisation occurs in the formation of a carious lesion, which will progress when the balance of factors favours demineralisation. Dental caries affects both the primary and permanent dentitions as well as the tooth crown and root, causing coronal caries and root caries, respectively. The main contributing factors for dental caries include the frequent consumption of high free sugar and insufficient exposure to fluoride.

### 2.3. Periodontal Disease

Periodontal disease is a chronic inflammatory disease that involves the periodontium, that is, the tissues surrounding and supporting the teeth, comprising gingiva, periodontal ligament, cementum and alveolar bone [22]. A pathogenic microbial biofilm, which accumulates at and below the gingival margin due to poor oral hygiene and inflicts destruction of the periodontium, is the key aetiological factor of periodontal disease, while tobacco smoking and diabetes are major risk factors. Severe periodontal disease, which is usually defined as the presence of a periodontal pocket (a pocket is formed by destruction of the periodontium and the subsequent loss of its attachment to the tooth) of greater than 6 mm depth, is regarded as a public health problem [2].

### 2.4. Inclusion Criteria

Cross-sectional, case–control, cohort and interventional studies, including randomised controlled trials and systematic reviews, published between January 2008 and April 2023 on the frequency of oral health visits and recall intervals and written in English were included regardless of age, sex and race of participants or country of publication.

### 2.5. Exclusion Criteria

Excluded from the review were studies conducted prior to January 2008, articles written in languages other than English, studies focusing on medically compromised individuals, case reports/case series, letters to editors, opinions, commentaries, reviews, conference abstracts, dissertations and theses.

### 2.6. Search Strategy

PubMed, Scopus and Embase databases were used to conduct an electronic search, which was made with only human control, without obtaining help from artificial intelligence (AI). Text words in titles, abstracts and index terms of articles were analysed after a preliminary search in PubMed and Embase. Thereafter, all identified key words and index terms were searched across the databases. The search strategy was developed with the support of a research librarian. The search terms included in the search strategy for PubMed are shown in Box 1. Specific terms for the other two databases were used to modify these terms where appropriate.

Box 1 **Search terms included in the PubMed search strategy.**

(‘Dental Health Services/utilization’ [Majr] OR ‘Dental Care/utilization’ [Majr] OR ‘Office Visits/utilization’ [Mesh] OR “Dental Visits, Frequency” [tiab:~0] OR “Dental Recall, Intervals” [tiab:~0]) AND (‘Periodontal Attachment Loss’ [Majr] OR ‘Gingival Diseases’ [Majr] OR ‘Dental Plaque’ [Mesh] OR ‘Dental Plaque Index’ [Mesh] OR ‘Dental Caries’ [Mesh] OR ‘Periodontal Index’ [Mesh] OR ‘DMF Index’ [Mesh]) AND (2008:2023[pdat]).

### 2.7. Study Selection

A preliminary review of the first 50 articles in alphabetical order was undertaken by two reviewers (N.A. and L.L.) to become familiar with the inclusion and exclusion criteria. Thereafter, a discussion between the two reviewers ensured that they reached a nearly perfect agreement (96%) in selecting studies with both reviewers ending up in the same decision, either in including or excluding 48 out of 50 articles, which had been undertaken for the preliminary review. The titles and abstracts of the studies were independently screened by the two reviewers and were excluded for their irrelevance to the current scoping review question. The reviewers discussed further and decided to seek the opinion of a third reviewer (D.B.) in case of any disagreement between the two reviewers during the selection process. The references were imported and managed by EndNote X9 bibliographic software.

## 3. Results

**Figure 1:**
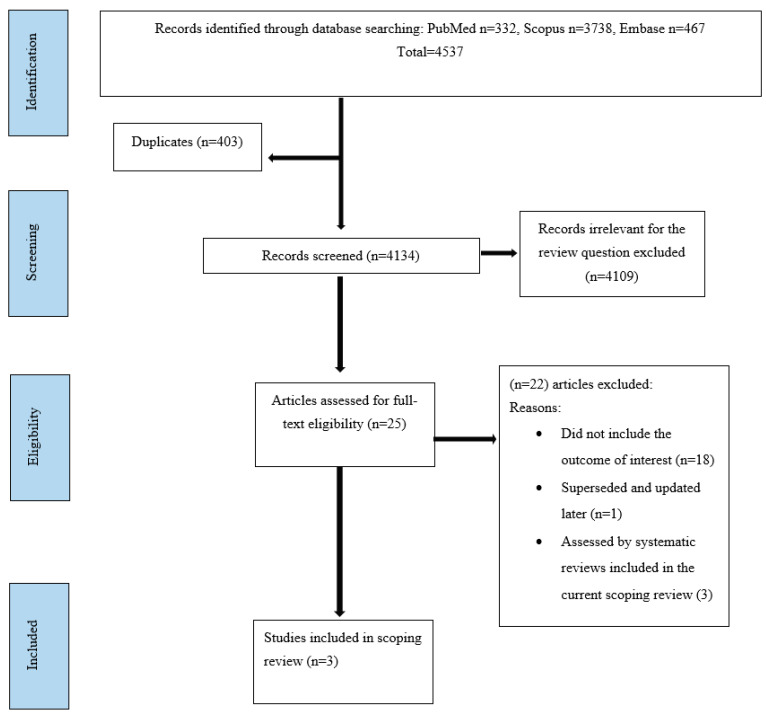
PRISMA flow diagram for the scoping review process.

A total of 4537 articles was yielded through the search strategy. The number of articles left for screening after removing duplicates was 4134. Having reviewed the titles and abstracts as per the inclusion and exclusion criteria, the reviewers decided to include 25 articles for full-text review after excluding 4109 articles, which were not in line with the review question. Subsequently, the reviewers carefully assessed these articles for their relevance and, after a lengthy discussion among all three reviewers, a consensus was reached to include three articles for full review. Figure 1 sums up the Preferred Reporting Items for Systematic reviews and Meta-analysis (PRISMA) [23] flowchart of the scoping review process alongside the number of articles excluded and the reasons for their exclusion.

Table 2 shows the basic characteristics of the studies included in the scoping review, such as objectives, methods, mode of analysis and results/conclusions/recommendations. The authors were from the UK (*n* = 2) and USA (*n* = 1). All three studies were systematic reviews, two of which were Cochrane reviews conducted in the UK, with oral health [20] and periodontal health [21] being the outcomes. The third review was from the USA and evaluated an appropriate recall interval for periodontal maintenance [22]. A brief description of these studies is provided below.

### 3.1. Cochrane Systematic Review on Recall Intervals for Oral Health in Primary Care Patients

Interestingly, this review was the third update of a Cochrane review on recall intervals for oral health in primary care patients, which was first published in 2005 [4] and later updated in 2007 [5] and 2013 [6]. Accordingly, this review [24] supersedes and updates the three previous versions of Cochrane reviews on this topic. All RCTs that included both children and adults receiving dental check-ups in primary care settings to compare recall intervals of any fixed length with different fixed-length intervals or recall intervals based on the clinician assessment of patient risk or no recall interval/patient-driven attendance (which may be symptomatic) up until January 2020 were included for the review. Of the 1423 studies retrieved after removing duplicates, 4 studies were chosen for full-text reading and, ultimately, 2 studies that met the selection criteria were included in the final review. One study compared 12-month versus 24-month recall intervals by measuring outcomes at 24 months, while the other compared the effects of 6-month, 24-month and risk-based recall intervals by measuring outcomes at 48 months. The data were extracted and risk of bias as well as the body of evidence were assessed using Cochrane Collaboration’s risk of bias tool and GRADE criteria, respectively. It was concluded that the evidence was of high certainty for adults attending dental check-ups in primary care settings to have little or no difference between risk-based and 6-month recall intervals in regard to dental caries, gingivitis and oral-health-related quality of life outcomes over a 4-year period. There was moderate- to high-certainty evidence for adults to have a little to no difference pertaining to dental caries, gingivitis and oral-health-related quality of life outcomes over a 4-year period when 24-month recall intervals were compared with 6-month or risk-based recall intervals. As such, they did not recommend further studies in this regard. However, since there was no certainty in the available evidence on recall intervals between dental check-ups for children and adolescents, the authors recommended well-designed RCTs of adequate duration with a sufficient number of participants for children and adolescents. They suggested conducting future studies to evaluate the potential harms of different recall intervals while using non-randomised studies to explore the effects of such recall intervals on mucosal/cancerous lesions and dento-facial development.

### 3.2. Cochrane Systematic Review on the Beneficial and Harmful Effects of Routine Scaling and Polishing (RSP) on Periodontal Health

This Cochrane review attempted to evaluate the beneficial and harmful effects of RSP on periodontal health when RSP is conducted at different recall intervals or RSP is performed by dentists as opposed to other dental care professionals. The review included RCTs of RSP that were conducted until January 2018, with a follow-up of at least six months, on healthy dentate adults who had no severe periodontitis. Comparisons included: planned RSP versus no scheduled RSP, RSP at a fixed recall interval versus different recall intervals and RSP performed by a dentist versus other dental care professional. Split-mouth trials and trials with participants who underwent specialist periodontal treatment or who were in the post-treatment maintenance phase were excluded. Data were extracted and risk of bias was assessed with the recommended Cochrane approach, while the certainty of evidence was assessed using the GRADE criteria. Two studies that provided data for two of the three comparisons (no study provided data for the comparison of dentist versus other dental professionals) were included for the final review. The authors concluded that there was a high certainty of evidence to suggest little or no difference in gingivitis, probing depths or quality of life over two to three years between RSP at 6 months and 12 months and no scheduled RSP, for adults without severe periodontitis who were routine attenders. Accordingly, they did not recommend further research that compares RSP at different recall intervals versus no scheduled RSP in adults who have no severe periodontitis and are regular dental attenders. However, the authors suggested conducting further studies that include clinical, patient-centred and economic outcomes to evaluate the effect and cost-effectiveness of periodontal care packages at the primary care level.

### 3.3. Systematic Review on the Appropriate Recall Interval for Periodontal Maintenance

This review assessed the evidence in regards to the most appropriate time interval for periodontal maintenance (PM) for adult patients previously treated for chronic periodontal disease, by including RCTs/controlled trials (CTs), retrospective studies, systematic reviews, review of systematic reviews and meta-analyses that were conducted until April 2014. Studies had to have measured at least one of the following outcomes—clinical attachment level, tooth retention or patient-based periodontal health assessments. Their search strategy yielded 1095 studies, of which 8 retrospective cohort studies evaluated the effect of compliance level with the recall intervals varying in the range of 3–6 months on tooth retention. Interestingly, there were no RCTs among these studies. The authors used the Critical Appraisal Skills Programme (CASP) guidelines that comprised 12 questions to evaluate the strength of studies and to synthesise the findings. According to the authors, this review was limited by the lack of published RCTs, uniform study designs and recall regimens. While concluding that the evidence was weak to support a fixed and specific PM recall interval for all patients, the authors were supportive of risk-based recall intervals over fixed or universal ones. They also recommended that future studies, including RCTs and large cohort studies, should aim to develop more evidence-based, customized and appropriate recall intervals.

## 4. Discussion

One of the three studies included in the current scoping review assessed the effectiveness of different frequencies of dental visits (recall intervals) on oral health, including dental caries and periodontal disease, while the focus of the other two studies was on the effect of such visits on periodontal disease/maintaining periodontal health. One study included RCTs involving both children and adults [24], while the other two studies [25,26] restricted the inclusion criteria to adults. Two of the reviews were Cochrane systematic reviews [24,25], which employed the recommended Cochrane approaches, such as the risk of bias tool in the Cochrane Handbook for Systematic Reviews of Interventions addressing six specific domains and the Grading of Recommendations Assessment, Development and Evaluation system (GRADE) criteria to assess the risk of bias and the certainty of evidence, respectively. Whilst these two reviews extracted data pertaining to the trial methods, participants, interventions and outcomes, the strategies to conduct meta-analysis, sensitivity analysis and the assessment of heterogeneity as well as publication bias were included where relevant. Conversely, the other review [26], which was the earliest published paper, used the Critical Appraisal Skills Programme (CASP) guidelines to evaluate the strength of the studies and to synthesize the findings in addition to extracting data in regards to population, interventions, design, outcomes and covariates, and assessing the heterogeneity of studies. Accordingly, information gleaned from these systematic reviews, particularly the two Cochrane systematic reviews [24,25] that included only RCTs, which are regarded to be at the top of the hierarchy of study designs pertaining to the strength of evidence provided, may have contributed considerably towards the overall body of evidence on the frequency of dental visits. This was further substantiated by the moderate-to-high certainty of evidence yielded by the two Cochrane systematic reviews. On the other hand, the authors of the other systematic review [26] were of the view that their review was hampered by the lack of RCTs and provided only weak evidence.

The limitations of our scoping review were confining the search strategy to English-only journal articles and excluding the grey literature, including theses, dissertations and conference proceedings. This, in turn, might have brought about a decrease in the number of studies included in the review. Nonetheless, we were able to minimise this to a certain extent by means of running the electronic search on three different databases and subsequently capturing a sizeable number of studies relevant to our scoping review question. In particular, the two Cochrane reviews included in our final review were comprehensive and encompassed almost all primary studies, specifically the RCTs and controlled clinical trials that fitted our scoping review question, while they imposed no restrictions on language, publication status or publication year of such studies. As such, we were satisfied that the current scoping review incorporated studies that were most pertinent and central to our research query.

## 5. Conclusions

Our scoping review retrieved more than 4000 studies, which were published throughout the study period in regards to different frequencies of dental visits. However, only three of these, which were the most relevant to the current review question and in line with the inclusion/exclusion criteria, were included in the final review. Our findings disclosed that the body of overall evidence was weak and of low quality for either fixed or universal frequency of dental visits, which are currently recommended to prevent dental caries and periodontal disease or maintain good oral health. For adults, there was little to no effect of making biannual, biennial or risk-based dental visits on dental caries and periodontal disease, which was supported by moderate- to high-certainty evidence according to the latest reports. As such, while indicating that further studies on the frequency of dental visits for adults are not required, our findings reiterate that dental professionals and dental insurance providers should make individually tailored, customised and risk-based recommendations for dental visits, in particular for adults, rather than encouraging fixed or universal frequencies of dental visits, including the common practice of recommending six-monthly dental visits. However, for children and adolescents, the available evidence on the effect of making different frequencies of dental visits on dental caries and periodontal disease is uncertain. Accordingly, further research involving children and adolescents on this issue necessitates well-designed randomised controlled trials (RCTs) and cohort studies of sufficient duration with an adequate number of participants.

### 5.1. Implications for Research

- Further studies are warranted for children and adolescents, in particular well-designed RCTs and large cohort studies of sufficient duration with an adequate number of participants and adjusted for risk assessment, to reflect a true difference among the varying frequencies of dental visits in regards to the potential beneficial and harmful effects on oral health.
- It is recommended that patient-centred factors as well as economic aspects should be incorporated as outcome measures, and the type of interventions should be clearly specified in future studies.
- Future studies should also focus on developing more evidence-based, customized and appropriate recall intervals.

### 5.2. Implications for Practice

- The available body of evidence indicates that firm conclusions cannot be made about the potential beneficial or harmful effects of different frequencies of dental visits on dental caries or periodontal disease, including the common practice of encouraging patients to make six-monthly dental visits. In this context, oral health professionals are suggested to make individually tailored, customised and risk-based recommendations for the frequencies of dental visits rather than encouraging fixed or universal frequencies of dental visits.

## Figures and Tables

**Table 1:** Global prevalence of dental caries and periodontal disease in 2019 according to WHO region.

WHO Region	Proportion of Persons with Untreated Dental Decay		Proportion of Persons with Severe Periodontal Disease
	Primary Dentition	Permanent Dentition	
African	38.6%	28.5%	22.8%
Eastern Mediterranean	45.1%	32.3%	17.4%
European	39.6%	33.6%	17.9%
Americas	43.2%	28.2%	18.9%
South-East Asia	43.8%	28.7%	20.8%
Western Pacific	46.2%	25.4%	16.3%
Global	42.7%	28.7%	18.8%

**Table 2:** Basic characteristics of the studies included in the scoping review.

Author/(Year)/ Country	Study Objectives	Method	Mode of Analysis	Results/Conclusions/Recommendations
Fee et al. [24] (2020)/UK	To determine the optimal recall interval for oral health in a primary care setting	A systematic review based on electronic searches of the following databases. The Cochrane Oral Health’s Trials Register (to 17 January 2020). The Cochrane Central Register of Controlled Trials (CENTRAL; 2019, Issue 12). MEDLINE via OVID (from 1946 to 17 January 2020). EMBASE via OVID (from 1980 to 17 January 2020). Inclusion criteria: Randomised controlled trials (RCTs). All children and adults receiving dental check-ups in primary care settings, notwithstanding their risk level for oral disease. Interventions: studies comparing recall intervals of any fixed length versus different fixed-length interval or recall intervals based on the clinician assessment of patient risk or no recall interval/patient-driven attendance (which may be symptomatic). No restrictions on language or publication date/status. Exclusion criteria: Studies other than RCTs. Studies with the type of check-up (which was different in each group) being compared. Outcome measures: Clinical status outcomes, including dental caries, periodontal disease, mucosal lesions, potentially malignant lesions, cancerous lesions and dento-facial development. Psychosocial outcomes, including oral-health-related quality of life and patient satisfaction with care provider/actual care received/appearance/oral comfort. Cost outcomes, including direct and indirect costs to the patient and health system. Other outcomes reported, including improved knowledge/attitudes in oral health and changes in dietary habits/any other oral-health-related behaviours and harms, such as fluorosis/overtreatment.	This Cochrane review adopted the below strategies in the analysis. Potential studies were independently assessed for eligibility by two review authors. All studies that met the inclusion criteria had undergone data extraction and assessment for risk of bias. Data pertaining to trial methods, participant characteristics, interventions and outcomes were extracted. The risk of bias was assessed using the risk of bias tool described in the Cochrane Handbook for Systematic review of Interventions. It was intended to conduct meta-analysis, sensitivity analysis or analysis of publication bias, if there was a sufficient number of studies. The body of evidence was assessed using GRADE criteria.	Results: The search strategy yielded 2289 references, which was brought down to 1423 after removing duplicates. Four studies were selected for full-text reading and of these only two studies met the inclusion criteria for final review. One study conducted in Norway compared 12-month versus 24-month recall intervals by measuring outcomes at 24 months. The other was conducted in the UK, which compared the effects of 6-month, 24-month and risk-based recall intervals by measuring outcomes at 48 months. The number of studies were not sufficient to conduct meta-analysis, publication bias or sensitivity analysis. Conclusions: The body of evidence emanating from the review was of high certainty for adults attending dental check-ups in primary care settings to have little or no difference between risk-based and 6-month recall intervals in regard to dental caries, gingivitis and oral-health-related quality of life outcomes over a 4-year period. There was moderate- to high-certainty evidence for adults to have a little to no difference pertaining to dental caries, gingivitis and oral-health-related quality of life outcomes over a 4-year period when 24-month recall intervals were compared with 6-month or risk-based recall intervals. For children and adolescents, there was no certainty in the available evidence on recall intervals between dental check-ups. Recommendations: As moderate- to high-certainty evidence was available pertaining to dental recall intervals for adults, the authors did not recommend further studies in this regard. Well-conducted RCTs with adequate sample sizes and duration are required for children and adolescents because of the uncertainty of evidence on recall intervals between dental-check-ups for them. Potential harms of different recall intervals, such as fluorosis and overtreatment, could be explored in future studies, while non-randomised studies are recommended to assess the effect of different recall intervals on dental outcomes, including mucosal/cancerous lesions and dento-facial development.
Lamont et al. [25] (2018)/UK	To ascertain the beneficial and harmful effects of: Routine scaling and polishing (RSP) on periodontal health; RSP at different recall intervals on periodontal health; RSP on periodontal health, when the treatment is provided by dentists compared with dental care professionals (dental therapists or dental hygienists).	This Cochrane systematic review was based on searching the following databases: Cochrane Oral Health’s Trials Register (to 10 January 2018). the Cochrane Central Register of Controlled Trials (CENTRAL) (the Cochrane Library, 2017, Issue 12). MEDLINE Ovid (from 1946 to 10 January 2018). Embase Ovid (from 1980 to 10 January 2018). Inclusion criteria: Randomised controlled trials (RCTs) of RSP incorporated with/without oral hygiene instructions (OHI), with a follow-up of at least six months. Healthy dentate adults who had no severe periodontitis and attended dental care in primary care settings. Interventions where RSP (with/without OHI) was provided by a dental professional with one or more of the following three comparisons: RSP at a planned, regular interval vs. no scheduled RSP for the duration of the trial. RSP at a planned, regular interval vs. RSP at a different planned, regular interval (e.g., every six months vs. every 12 months). RSP provided by a dentist at a planned, regular interval vs. RSP provided by a dental hygienist or dental therapist at the same planned, regular interval. Exclusion criteria: Split-mouth trials. Participants with severe periodontitis. Participants that underwent specialist periodontal treatment and in the post-treatment maintenance phase. Outcome measures: Primary outcome was periodontal disease ascertained by gingival indices, whereas secondary outcomes included clinical status, patient-centred and economic cost factors.	The following strategies were employed in the analysis: Two review authors independently assessed the studies for eligibility and extracted data to assess for risk of bias. All studies that met the inclusion criteria underwent data extraction and assessment for risk of bias. Data pertaining to study designs, participant characteristics, interventions and outcomes were recorded. The risk of bias was assessed using the recommended Cochrane approach with a two-part tool addressing six specific domains. Meta-analysis, sensitivity analysis, clinical heterogeneity and analysis of publication bias were planned, provided there was a sufficient number of studies. GRADE criteria were used to analyse the certainty of the evidence.	Results: The search strategy retrieved 1002 records after removing duplicates. Abstract and title sifting resulted in only one study being retained, while another study from the previous review was included, making it two studies for the final review. Both studies were based in the UK and included 1711 participants who did not have severe periodontitis attending regularly at general dental practices. These two studies provided data for two of the three comparisons intended—none of the studies had data for the third comparison. The outcomes were measured at 24 months in one study and at 36 months in the other. Conclusions: There was a high certainty of evidence to suggest little or no difference in gingivitis, probing depths or quality of life over two to three years between RSP at 6 month, 12 month and no scheduled RSP, for adults without severe periodontitis who were routine attenders. There was low-certainty evidence to suggest little or no difference in plaque levels over two years. Although there was high-certainty evidence for RSP at 6 months versus at >12 months to reduce calculus over two- to three-year follow-up clinical relevance of such small reductions was uncertain. While not evaluating the harmful effects of RSP, these studies provided very low-certainty evidence for the cost of RSP. Recommendations: The authors did not recommend further studies comparing RSP considering that there was high-certainty evidence for RSP at 6 or 12 months versus no scheduled RSP to significantly reduce periodontal disease measured by gingival indices over three years in regularly attending adults with no severe periodontitis. The authors recommended further research on the effects and cost-effectiveness of interventions, including periodontal care packages that combine advice, recommendations for oral care products and scaling and polishing at the primary dental care level to manage moderate-to-severe periodontal disease. Such studies should measure clinical status, patient-centred and economic cost factors as outcomes.
Farooqi et al. [26] (2015)/USA	To evaluate the evidence regarding the most appropriate time interval for periodontal maintenance (PM), for patients previously treated for chronic periodontal disease	A search was conducted in MEDLINE, EMBASE and PubMed up to April 2014. Inclusion criteria: RCTs/controlled trials (CTs), retrospective studies, systematic reviews, review of systematic reviews and meta-analyses. Study population included solely or primarily adults with verified periodontal disease. Periodontal disease diagnosis that was consistent with the 1990 International Workshop for the Classification of Periodontal Disease and Conditions. Studies that had defined PM procedures and were consistent with the broad understanding of supportive periodontal therapy undertaken after successful active periodontal therapy. Studies that included at least one of the following outcomes: > Clinical attachment level. > Tooth retention. > Patient-based assessments of periodontal health. Exclusion criteria: Narrative reviews and other studies not pertinent to the inclusion criteria.	The strength of studies was evaluated and the findings were synthesized as per the Critical Appraisal Skills Programme (CASP) guidelines that comprised 12 questions.	Results: The search strategy yielded 1095 articles, of which 8 cohort studies were included in the final review. There were no RCTs among them. The effect of compliance level with the suggested PM regimen, varying in the range of 3–6 months, on tooth retention as the outcome was evaluated by the eight studies included. While a considerable heterogeneity among the studies existed, one study was rated as excellent, three as good and two each as fair and poor, according to the quality and strength of the studies rated using the CASP criteria. The main findings of the review were as follows: Regular compliers with more frequent PM recall intervals (3–6 months) had fewer teeth extracted than irregular compliers. There was no statistically significant difference between regular and irregular compliers when the PM interval was <6 or >6 months. The patients who are highly compliant (attended at least 70% of 3–4 monthly PM visits) were more likely to lose teeth than those who attended less than 70%. Conclusions: While highlighting that the review was limited by the non-availability of published RCTs on this subject, studies directly comparing different recall intervals and their effect on periodontal parameters or tooth loss, information on non-compliers (barring one study) and uniform study designs and recall regimens, the authors concluded that: There was weak evidence to support a fixed and specific PM recall interval. More frequent PM recall visits were favoured by mixed evidence, while the optimum frequency was unclear. Risk-based recommendations for recall intervals were preferred over fixed or universal ones. Recommendations: Further studies, including RCTs with standardised designs and large longitudinal cohort studies with varying recall intervals and accounting for risk assessment to evaluate the effects of varying intervals on the stability of the periodontium, are required. Such studies should aim to develop more evidence-based, customized and appropriate recall intervals.

## Data Availability

No new data were generated.
